# Hypoxia Modulates Regenerative Potential of Fetal Stem Cells

**DOI:** 10.3390/app12010363

**Published:** 2021-12-30

**Authors:** Yixuan Amy Pei, Ming Pei

**Affiliations:** 1 Stem Cell and Tissue Engineering Laboratory, Department of Orthopaedics, West Virginia University, Morgantown, WV 26506, USA; 2 Perelman School of Medicine, University of Pennsylvania, Philadelphia, PA 19104, USA; 3 WVU Cancer Institute, West Virginia University, Morgantown, WV 26506, USA

**Keywords:** fetal stem cell, hypoxia, differentiation, fetal nucleus pulposus cell, fetal synovium-derived stem cell

## Abstract

Adult mesenchymal stem cells (MSCs) are prone to senescence, which limits the scope of their use in tissue engineering and regeneration and increases the likelihood of post-implantation failure. As a robust alternative cell source, fetal stem cells can prevent an immune reaction and senescence. However, few studies use this cell type. In this study, we sought to characterize fetal cells’ regenerative potential in hypoxic conditions. Specifically, we examined whether hypoxic exposure during the expansion and differentiation phases would affect human fetal nucleus pulposus cell (NPC) and fetal synovium-derived stem cell (SDSC) plasticity and three-lineage differentiation potential. We concluded that fetal NPCs represent the most promising cell source for chondrogenic differentiation, as they are more responsive and display stronger phenotypic stability, particularly when expanded and differentiated in hypoxic conditions. Fetal SDSCs have less potential for chondrogenic differentiation compared to their adult counterpart. This study also indicated that fetal SDSCs exhibit a discrepancy in adipogenic and osteogenic differentiation in response to hypoxia.

## 1. Introduction

Adult mesenchymal stem cells (MSCs) are a potential solution for cell-based tissue engineering and regeneration due to their self-regenerative capacity and potential to differentiate into a multitude of cell types [1]. However, many challenges exist. Given the fact that most patients are elderly, their stem cells are inherently senescent. Moreover, a large quantity of autologous adult stem cells is required for tissue engineering—a process that necessitates long-term ex vivo expansion, which can cause cell senescence, resulting in lowered plasticity and limited proliferation capacity [2]. These challenges restrict the clinical application of adult MSCs despite their well-characterized properties [3].

In comparison to adult MSCs, fetal MSCs have greater differentiation potential, lower immunogenicity, longer telomeres, and increased intrinsic homing and engraftment ability [4,5]. Despite their immense potential for regenerative medicine, given ethical concerns [6], there exists limited knowledge regarding the regenerative potential of fetal MSCs and their responses to external environment stimulation, which have been well-characterized in adult MSCs, such as decellularized extracellular matrix (dECM) [7], hypoxia, and soluble factors such as basic fibroblast growth factor [8,9]. Our previous report found that, as an alternative to adult MSCs, which experience replicative senescence, human fetal synovium-derived stem cells (SDSCs) from passage 9 exhibited higher chondrogenic potential than those from passage 2 [10], indicating that fetal MSCs might have different regenerative potentials compared to adult MSCs. We also found that expansion on dECM could modulate the chondrogenic potential of human fetal SDSCs [10,11], suggesting that the external environment can adjust fetal cell biological properties for regenerative purposes. There are few articles investigating the influence of hypoxia on the regenerative potential of fetal stem cells despite the importance of hypoxia in adult stem cell differentiation [12].

In this study, two types of fetal cells with chondrogenic potential [13], SDSCs (a tissue-specific stem cell for chondrogenesis) [14] and nucleus pulposus cells (NPCs, a chondrogenic progenitor cell) [15], were used to characterize the influence of hypoxia in both the cell expansion (differentiation potential) and chondrogenic induction phases (degree of differentiation). Hypoxia is critical for chondrogenesis [16], given that articular cartilage is an avascular tissue with oxygen levels of about 1–5% [17]. Furthermore, increasing evidence indicates that hypoxia plays a role in the adipogenesis and osteogenesis of adult MSCs [18] and the inherently hypoxic stem cell niche facilitates signal transduction [19]; however, we still lack knowledge regarding the influence of hypoxia on the adipogenic and osteogenic differentiation of fetal cells. In addition to clarifying the complex interplay of hypoxia and fetal stem cells in an in vitro cell culture environment, the findings of this study will also facilitate our understanding of the crosstalk between chondrogenesis and adipogenesis [20], as well as osteogenesis and adipogenesis [21].

## 2. Materials and Methods

### 2.1. Human Fetal NPC and SDSC Culture

Human fetal NPCs and SDSCs [10,11] (ScienCell Research Laboratories, Carlsbad, CA, USA) were plated into T175 plastic flasks at 3000 cells/cm^2^ in growth medium (_*α*_-minimum essential medium (*α*-MEM) containing 10% fetal bovine serum (FBS), 100 U/mL penicillin, 100 μg/mL streptomycin, and 0.25 μg/mL fungizone) (Thermo Fisher Scientific, Waltham, MA, USA). The cells were cultured in a 5% CO_2_ incubator at 37 ^◦^C in either hypoxia (5% O_2_, 38 mm Hg) or normoxia (21% O_2_, 159 mm Hg). The medium was changed every other day. During expansion, cell number was measured and morphology was documented.

**Figure 1.**
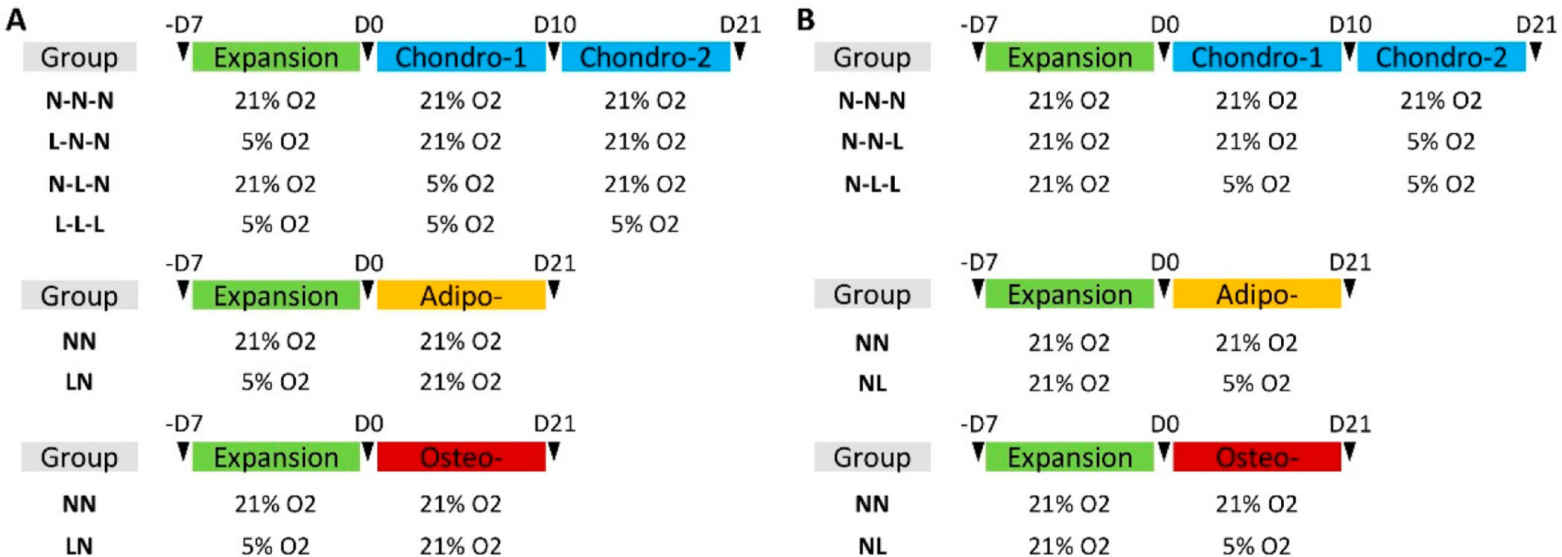
Schematic of Experimental Design. Both fetal NPCs and SDSCs were expanded in T175 plastic flasks for 7 days followed by either two phases of chondrogenic differentiation (D0-D10, D10-D21) in a pellet culture system, or one phase of either adipogenic or osteogenic differentiation(D0-D21) in T25 plastic flasks. (**A**) The pretreatment culture regimen was designed to subject each chondrogenic cell type to four treatment conditions that vary in oxygen tension (N-N-N, L-N-N, N-L-N, and L-L-L). Adipogenic and Osteogenic cells were subject to hypoxia or normoxia during expansion followed by differentiation induction in normoxia (LN and NN). (**B**) The differentiation treatment regimen was designed such that chondrogenic cells were expanded in normoxia and then subjected to three treatment conditions varying in oxygen tension (N-N-N, N-N-L, and N-L-L). Adipogenic and osteogenic cells were subjected to normoxia in cell expansion followed by hypoxia or normoxia during differentiation (NL and NN). Incubators were set to N = normoxia (21% O_2_) incubation and L = hypoxia (5% O_2_) incubation.

Two primary designs were utilized in this experiment: (1) Pretreatment culture regimen (Figure 1A): passage 3 human fetal NPCs and SDSCs were grown for 7 days in either hypoxia or normoxia. Afterward, cells were reseeded into T25 plastic flasks for 21 days of either adipogenic or osteogenic induction (LN and NN) or centrifuged to form pellets for chondrogenic induction. The pellets from NPCs or SDSCs that were pretreated under normoxia were allowed to grow in normoxia D0-D21 as a control (N-N-N) and in hypoxia D0-D10 and transferred to normoxia D10-D21 (N-L-N). The pellets that were pretreated under hypoxic conditions were allowed to grow in normoxia D0-D21 (L-N-N) and in hypoxia D0-D21 (L-L-L); and (2) Differentiation treatment regimen (Figure 1B): passage 3 human fetal NPCs and SDSCs were expanded for 7 days in normoxia. Afterward, cells were incubated in T25 plastic flasks for 21-day adipogenic or osteogenic induction in either normoxia (NN) or hypoxia (NL). Pellets were subsequently formed and separated into three treatment groups: normoxia for D0-D21 (N-N-N), hypoxia for D0-D21 (N-L-L), and normoxia from D0-D10 and then transferred to hypoxia from D10-D21 (N-N-L).

### 2.2. Evaluation of Proliferation, Surface Markers, and Stemness Genes

#### 2.2.1. Cell Proliferation

Levels of expanded cell proliferation in either normoxia or hypoxia were assessed using Click-iT 5-ethynyl-2^′^-deoxyuridine (EdU) Cell Proliferation Assay kit (Thermo Fisher Scientific). Cells reaching 50% confluence were incubated in the medium with 10 μM EdU at 37 °C for 18 h. Following fixation in 4% paraformaldehyde (Thermo Fisher Scientific), cells (2 × 10^5^ each group) were treated with Click-iT^®^ reaction cocktail for 30 min and fluorescence was evaluated by a FACS Calibur (BD Biosciences, San Jose, CA, USA) using the FCS Express software package (De Novo Software, Los Angeles, CA, USA).

#### 2.2.2. Surface Phenotypes of Expanded Cells

Cell surface marker expression was evaluated using the following primary antibodies: the stage-specific embryonic antigen 4-PE (SSEA4-PE; BioLegend, Dedham, MA, USA), CD73-APC (Thermo Fisher Scientific), CD90-APC-Vio770 (Miltenyi Biotec, San Diego, CA, USA), CD105-PerCP-Vio700 (Miltenyi Biotec), and CD146-PE (Thermo Fisher Scientific). Expanded cells (2 × 10^5^ each group) were first blocked through a 30 min incubation in cold phosphate-buffered saline (PBS) containing 0.1% ChromPure Human IgG whole molecule (Jackson ImmunoResearch Laboratories, West Grove, PA, USA) followed by incubation with primary antibodies at 4 °C for 30 min. Fluorescence was evaluated by a FACS Calibur (BD Biosciences) using the FCS Express software package (De Novo Software).

#### 2.2.3. Reverse Transcription Quantitative Polymerase Chain Reaction

TRIzol^®^ reagent (MilliporeSigma, Burlington, MA, USA) was used to extract total RNA from each sample (*n* = 4). Two μg of RNA was used for reverse transcription with a High-Capacity cDNA Reverse Transcription Kit (Thermo Fisher Scientific). Stemness genes for evaluation included *NANOG*, *SOX2*, *KLF4*, *BMI1*, *MYC*, *NOV*, *POU5F1*, and *NES*. Other genes for assessment included: chondrogenesis genes (*SOX9*, *ACAN*, *COL2A1*, *PRG4*, *COL10A1*, *COL1A1*, *FOXF1*, *FBLN1*, *CDH2*, and *FN1*), adipogenesis genes (*LPL*, *PPARG*, *FABP4*, and *CEBPA*), and osteogenesis genes (*RUNX2*, *SPP1*, *BGLAP*, and *SP7*). The relevant primers were obtained from Applied Biosystems (Thermo Fisher Scientific) as part of the Custom TaqMan^®^ Gene Expression Assays. *GAPDH* was carried out as the endogenous control gene. Assay IDs of primers were listed in Table 1. Reverse transcription quantitative polymerase chain reaction (RT-qPCR) was performed using Applied Biosystems^™^ 7500 Fast Real-Time PCR System (Thermo Fisher Scientific). Relative transcript levels were calculated as χ = 2^−ΔΔCt^, in which ΔΔCt = ΔE−ΔC, ΔE = Ct_exp_−Ct_GAPDH_, and ΔC = Ct_ct1_−Ct_GAPDH_.

### 2.3. Three-Lineage Differentiation

#### 2.3.1. Chondrogenic Induction and Evaluation

To induce chondrogenic differentiation, pellet models were formed by centrifuging an aliquot of 0.4 × 10^6^ expanded cells at 500× *g* for 7 min in a 15-mL polypropylene tube. Pellets were then cultured in a serum-free high-glucose Dulbecco’s Modified Eagle’s Medium (DMEM) with 100 U/mL penicillin, 100 μg/mL streptomycin, 40 μg/mL proline (MilliporeSigma), 100 nM dexamethasone (MilliporeSigma), 0.1 mM ascorbic acid-2-phosphate (Thermo Fisher Scientific), ITS^™^ Premix (BD Biosciences), and 10 ng/mL transforming growth factor beta 3 (TGFβ3; PeproTech, Rocky Hill, NJ, USA). The pellets were collected at days 0, 10, and 21 for histology, immunohistochemistry, and RT-qPCR (see Section 2.2.3 for details).

For histology, representative pellets (*n* = 3) were fixed overnight in 4% paraformaldehyde at 4 ^◦^C, followed by dehydrating through a gradient ethanol series, clearing with xylene, and embedding in paraffin blocks. Then, 5 μm sections of the pellets were stained with Alcian blue (MilliporeSigma) (counterstained with fast red) for sulfated glycosaminoglycans (GAGs). For immunohistochemistry, the sections were immunolabeled with primary antibodies against type II collagen (catalog number II-II6B3; Developmental Studies Hybridoma Bank (DSHB), Iowa City, IA, USA) and type I collagen (catalog number GTX26308; DSHB), followed by the secondary antibody of biotinylated horse anti-mouse IgG (Vector, Burlingame, CA, USA). Immunoactivity was detected using Vectastain ABC reagent (Vector) with 3,3^′^-diaminobenzidine as a substrate.

#### 2.3.2. Adipogenic Differentiation and Evaluation

Expanded cells around 95% confluence underwent a 21-day incubation in adipogenic medium (α-MEM supplemented with 10% FBS, 1 μM dexamethasone, 0.5 mM isobutyl1-methyxanthine (Thermo Fisher Scientific), 200 μM indomethacin (MilliporeSigma), and 10 μM insulin (BioVendor, Asheville, NC, USA)). In order to stain intracellular lipid-filled droplets, samples (*n* = 4) were fixed in 4% paraformaldehyde and incubated with a 0.6% (*w*/*v*) Oil Red O (ORO) solution (60% isopropanol, 40% water) for 10 min. Adipogenic marker genes were quantified using RT-qPCR (see Section 2.2.3 for details).

For the assessment using western blot, protein extraction was performed by suspending cells in ice-cold lysis buffer (Cell Signaling Technology, Inc., Danvers, MA, USA) with protease inhibitors (Thermo Fisher Scientific). Total protein quantification was performed using Pierce^™^ BCA Protein Assay Kit (Thermo Fisher Scientific). Thirty μg of protein from each sample were separated using NuPAGE^™^ Bis-Tris Mini Gels (Thermo Fisher Scientific) in the XCell SureLock^™^ Mini-Cell (Thermo Fisher Scientific) at 120 V for 15 min and 160 V for 1 h. Bands were transferred onto a nitrocellulose membrane using an XCell II^™^ Blot module (Thermo Fisher Scientific) at 30 V at 4 ^◦^C overnight (16 h). The membrane was incubated with a primary monoclonal antibody targeting FABP4 (catalog number sc-271529, Santa Cruz Biotechnology, Dallas, TX, USA) and internal control GAPDH (catalog number AM4300, Thermo Fisher Scientific) in 5% bovine serum albumin fraction V (BSA) in TBST buffer (10 mM Tris–HCl, pH 7.5, 150 mM NaCl, 0.05% Tween-20) at 4 ^◦^C overnight, followed by the secondary antibody of horseradish peroxidase-conjugated goat anti-mouse (Thermo Fisher Scientific) for 30 min. ECL^™^ Prime Western Blotting Detection Reagents (Amersham Biosciences, Waltham, MA, USA) were used for exposure.

#### 2.3.3. Osteogenic Differentiation

Expanded cells around 95% confluence were incubated for 21 days in osteogenic medium containing DMEM, 10% FBS, 100 U/mL penicillin, 100 μg/mL streptomycin, 50 μg/mL L-ascorbic acid, 100 nM dexamethasone, and 10 mM β-glycerol phosphate (Thermo Fisher Scientific). For evaluation of matrix mineralization, induced cells (*n* = 4) were fixed with 70% ice-cold ethanol for 1 h and then incubated in 1% Alizarin Red S (ARS) solution (pH = 4.3; MilliporeSigma) for 20 min at room temperature with agitation. After rinsing three times with PBS, images of calcium deposition were taken using an Olympus IX51 microscope (Olympus America Inc., Center Valley, PA, USA). Osteogenic marker genes were quantified using RT-qPCR (see Section 2.2.3 for details).

### 2.4. Statistical Analysis

The Mann–Whitney U test was used for pairwise comparison. All statistical analyses were performed with SPSS 20.0 statistical software (SPSS Inc., Chicago, IL, USA). A *p*-value of less than 0.05 is considered statistically significant.

## 3. Results

### 3.1. Effects of Hypoxia on Proliferation Potential, Surface Marker Expression, and Stemness Gene Expression of Fetal Cells

**Figure 2.**
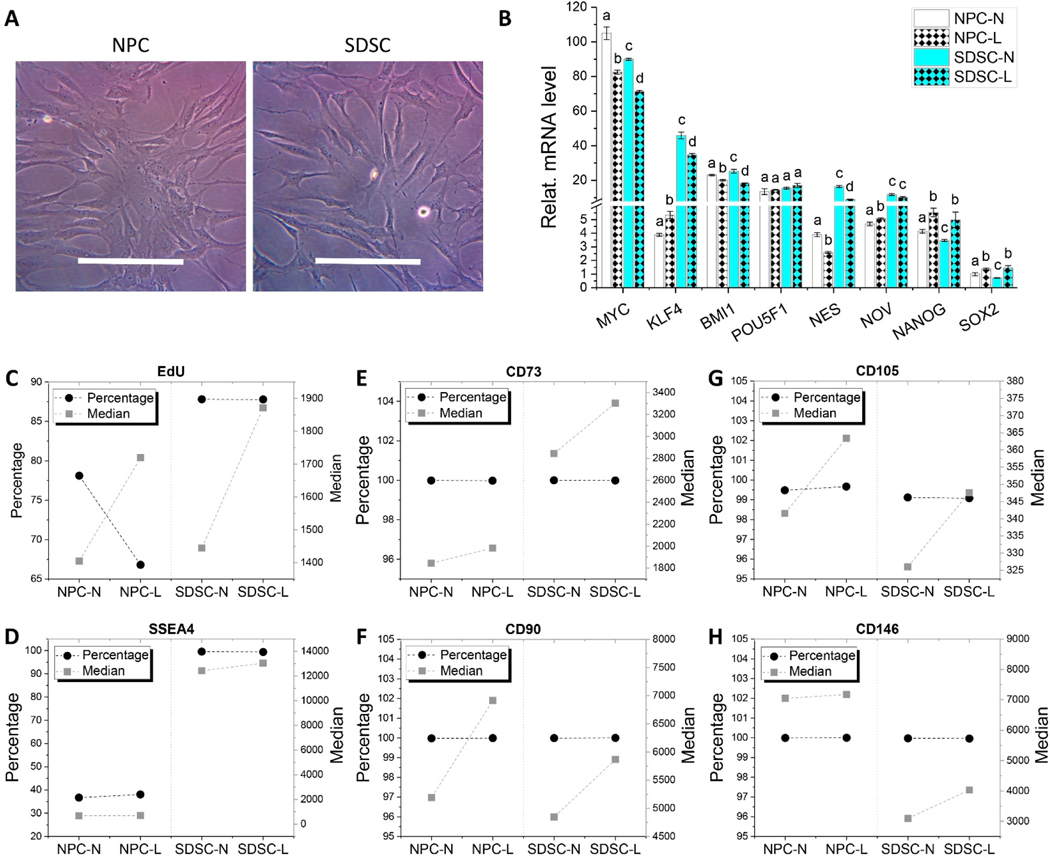
Evaluation of stemness gene expression, proliferation potential, and surface marker expression in fetal cells following either normoxia (N) or hypoxia (L) incubation. (**A**) Fetal NPC and SDSC cell morphology during expansion. (**B**) RT-qPCR assessed the expression of stemness genes (*MYC*, *KLF4*, *BMI1*, *POU5F1*, *NES*, *NOV*, *NANOG*, and *SOX2*) in both fetal cell types after hypoxia treatment, normalized against *GAPDH* levels as an internal control. Data (*n* = 4) are represented in bar charts. Different letters indicate a statistically significant difference compared to the groups within the same gene type (*p* < 0.05). Flow cytometry assessed the positive percentage and median intensity of relative EdU incorporation (**C**) and expression of MSC surface markers SSEA4 (**D**), CD73 (**E**), CD90 (**F**), CD105 (**G**), and CD146 (**H**).

Despite no significant differences in cell morphology between NPCs and SDSCs during expansion (Figure 2A), RT-qPCR data showed a discrepancy in stemness gene expressions of these two human fetal cells and their response to hypoxia (Figure 2B). In brief, fetal NPCs exhibited higher expression of *MYC*, *NANOG*, and *SOX2* than fetal SDSCs whereas fetal SDSCs displayed higher expression of *KLF4*, *NES*, and *NOV* than fetal NPCs. Interestingly, hypoxia decreased *MYC*, *BMI1*, and *NES* and increased *NANOG* and *SOX2* in both fetal cell types.

Relative EdU incorporation data (Figure 2C) and SSEA4 expression (Figure 2D) indicated that SDSCs exhibited higher proliferation capacity than NP cells, both of which increased in median intensity after hypoxia treatment. Flow cytometry data also showed that SDSCs exhibited higher expression of CD73 (Figure 2E) despite lower expression of CD90 (Figure 2F), CD105 (Figure 2G), and CD146 (Figure 2H) in median intensity compared to NPCs; interestingly, hypoxia increased median intensity of the above CD markers of both fetal cells to some degree compared to their normoxic counterparts.

### 3.2. Effects of Hypoxia Pretreatment on Chondrogenic Capacity of Fetal Cells

**Figure 3.**
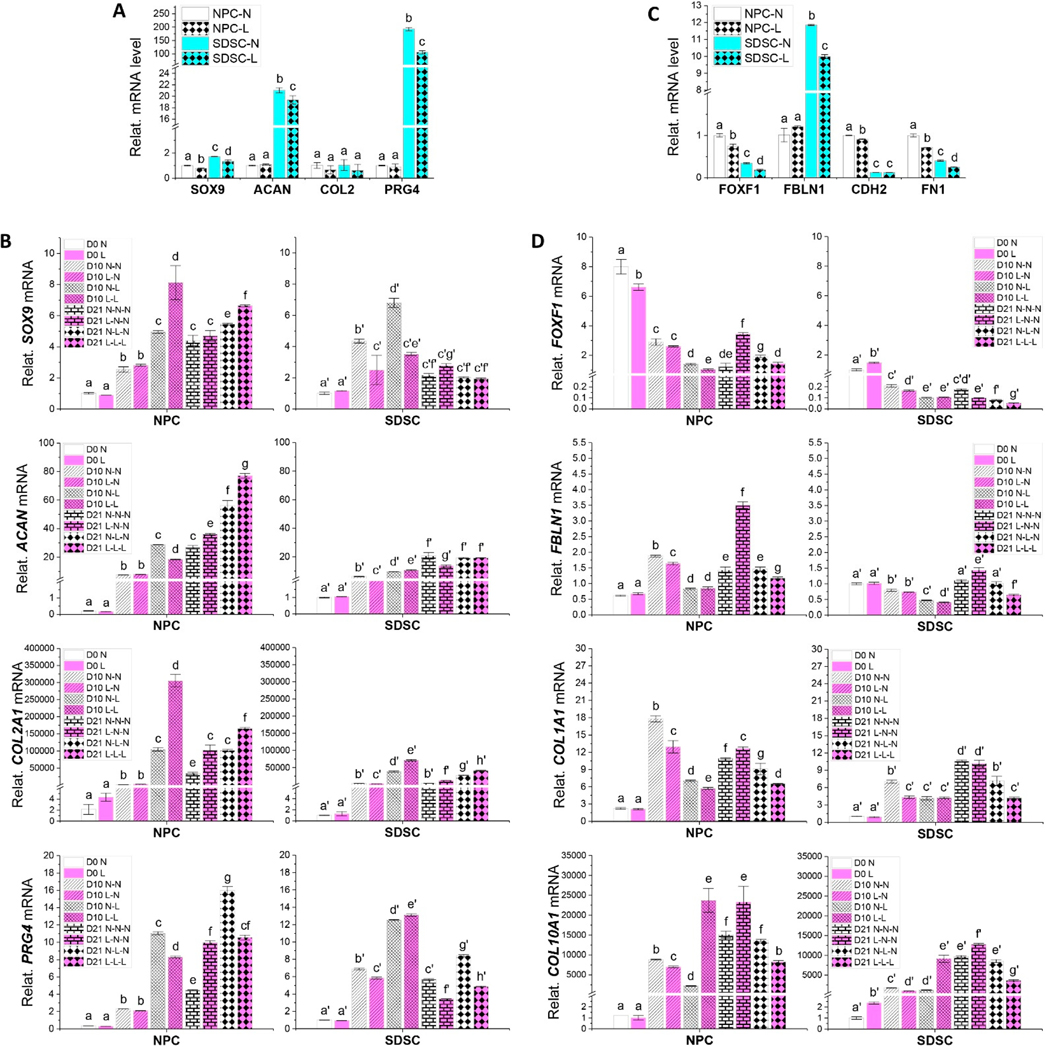
Evaluation of the effect of hypoxia pretreatment on chondrogenic potential of fetal MSCs using RT-qPCR. Passage 4 NPCs and SDSCs were grown to confluence in growth medium in either the normoxia (N) or hypoxia (L) environment, which were referred to as cell samples (before induction). 0.4 × 10^6^ cells were centrifuged to form pellets, which were then incubated in growth medium overnight before chondrogenic induction; these pellets were referred to as chondrogenic day 0 pellets (D0). Day 10 (D10) and Day 21 (D21) pellets were collected during 21-day chondrogenic induction with varied hypoxia treatment. Assessment of key chondrogenic marker genes *SOX9*, *ACAN*, *COL2A1*, and *PRG4* before (**A**) and after chondrogenic induction (**B**). Chondrogenic-associated genes *FOXF1*, *FBLN1*, *CDH2*, and *FN1* were also assessed before (**C**) and after chondrogenic induction (**D**), normalized against *GAPDH* levels as an internal control. Data (*n* = 4) are represented in bar charts. Different letters indicate a statistically significant difference compared to the groups within the same cell type (*p* < 0.05).

To determine the influence of hypoxia on chondrogenic gene expression of both fetal cells, major chondrogenic marker genes including SOX9, *ACAN, COL2A1*, and PRG4 were evaluated using RT-qPCR. The data showed that hypoxia decreased expression of *ACAN* and *PRG4* in SDSCs despite there being no significant difference in NPCs (Figure 3A). After 21-day chondrogenic induction in a pellet culture system, we found that hypoxia pretreatment significantly increased chondrogenic marker gene (*ACAN, COL2A1*, and *PRG4*) expression in NPCs compared to the normoxia group (L-N-N versus N-N-N) despite nonsignificant expression of SOX9 (Figure 3B). Interestingly, exposure to low oxygen in the early phase of chondrogenic induction (D0-D10) significantly increased *SOX9, ACAN*, and *PRG4* expression compared to the hypoxia pretreatment group (N-L-N versus L-N-N) (Figure 3B). We also found that hypoxia treatment throughout both the expansion and differentiation phases (L-L-L) yielded NPCs with the highest expression of *SOX9, ACAN*, and *COL2A1* (Figure 3B).

SDSCs responded to hypoxia differently compared to NPCs (Figure 3B). We found that hypoxia pretreatment increased *SOX9* and *COL2A1* expression but decreased *ACAN* and *PRG4* levels compared to normoxia treatment. Exposure to low oxygen in the early phase of chondrogenic induction (D0-D10) promoted *COL2A1* and *PRG4* compared to the normoxia group and hypoxia preconditioned groups (N-L-N versus N-N-N/L-N-N), which may have significance clinically given the fact that *PRG4* encodes lubricin, a secreted protein that helps in joint lubrication and movement. Hypoxia treatment throughout both the expansion and differentiation phases (L-L-L) yielded SDSCs with the highest expression of *COL2A1*. Overall, SDSCs exhibited a weaker response to chondrogenic induction than NPCs in both normoxic and hypoxic conditions.

We also assessed other chondrogenesis-associated gene expressions in response to hypoxia pretreatment, including *FBLN1* (articular cartilage marker gene), *FOXF1* (NPC marker gene), *CDH2* (mesenchymal condensation marker gene), and *FN1* (cell condensation associated gene) (Figure 3C). We found that NPCs exhibited higher expression of *FOXF1* while SDSCs displayed higher expression of *FBLN1*, which is in line with previous reports that *FOXF1* was highly expressed in human NP while *FBLN1* was advantageously expressed in human articular cartilage [22]; SDSCs are a tissue-specific stem cell for chondrogenesis [14]. NPCs also exhibited significant expression of *CDH2* and *FN1* compared to SDSCs, indicating the greater chondrogenic potential of NPCs [23,24]. Interestingly, hypoxia pretreatment decreased most of these condensation gene expressions, particularly for SDSCs. After 21-day chondrogenic induction, we found that hypoxia pretreatment of NPCs exhibited the highest expression of *FOXF1* and *FBLN1* as well as *COL1A1* and *COL10A1* while SDSCs displayed the highest expression of *FBLN1* as well as *COL10A1* (Figure 3D). Interestingly, hypoxia treatment throughout both the expansion and differentiation phases (L-L-L) significantly decreased early chondrogenic marker *COL1A1* and hypertrophic marker *COL10A1* expression in both NPCs and SDSCs.

**Figure 4.**
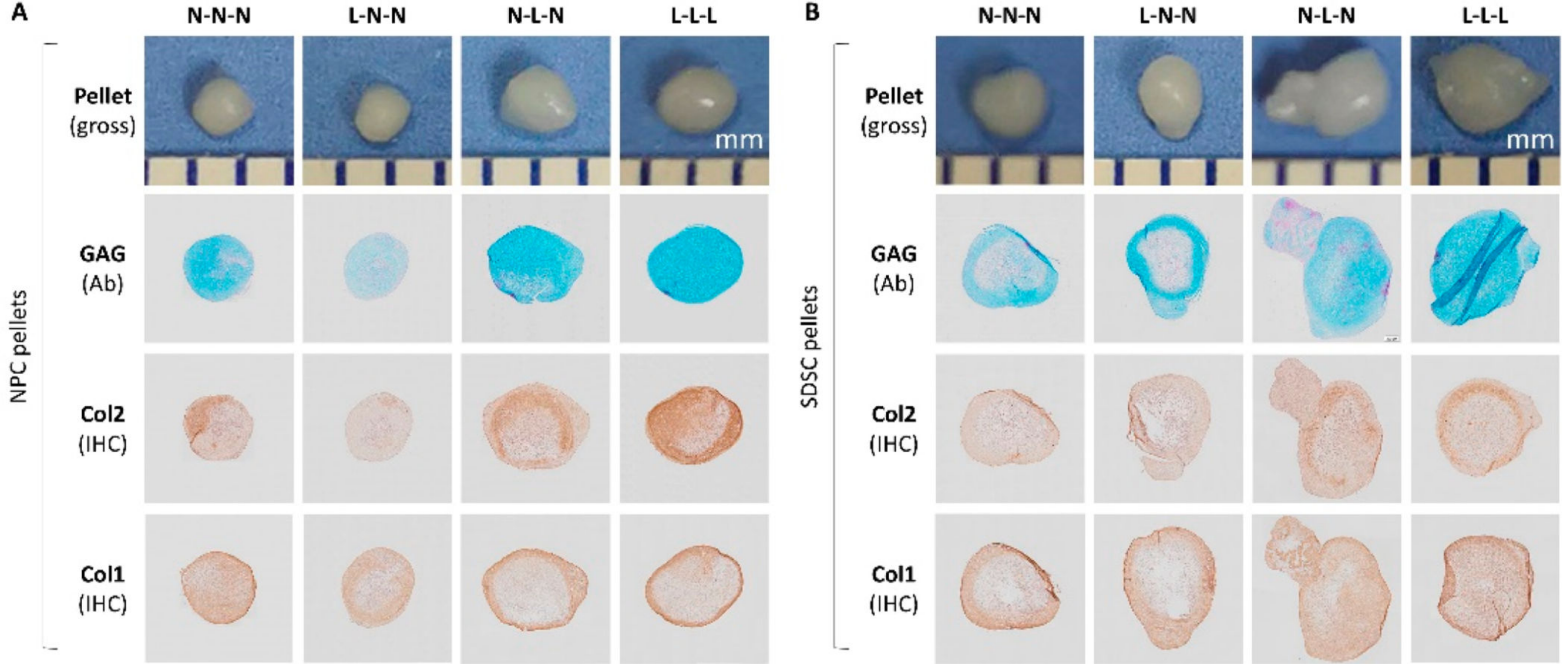
Evaluation of the effect of hypoxia pretreatment on the chondrogenic potential of fetal MSCs using histology and immunohistochemical staining. NPC (**A**) and SDSC (**B**) pellets were cultured in chondrogenic induction medium for 21 days. Hypoxia was used in cell expansion and subsequent induction in different combinations (see Figure 1A). Histology using Alcian blue staining (Ab) for sulfated GAG (with fast red as a counterstain) and immunohistochemical staining (IHC) for type II collagen (Col2) and type I collagen (Col1) (with hematoxylin as a counterstain) along with a gross photo to indicate the size of the pellet (mm).

Histologically, NPC pellets were more dense in Alcian blue staining (Ab) for sulfated GAG and immunohistochemical staining (IHC) for type II collagen compared to SDSC pellets with a larger size, particularly for the hypoxia treatment group (L-L-L) (Figure 4A versus Figure 4B), which is in line with the above RT-qPCR data. Intriguingly, the hypoxia pretreatment group yielded NPC pellets with the weakest staining of sulfated GAG and type II collagen (Figure 4A). SDSC pellets with larger size and more dense staining were found in the treatment groups by either normoxia in expansion followed by hypoxia in early chondrogenic induction (D0-D10) (N-L-N) or hypoxia throughout both the expansion and chondrogenic differentiation phases (L-L-L) (Figure 4B).

### 3.3. Effects of Hypoxia Pretreatment on Adipogenic and Osteogenic Potentials of Fetal Cells

**Figure 5.**
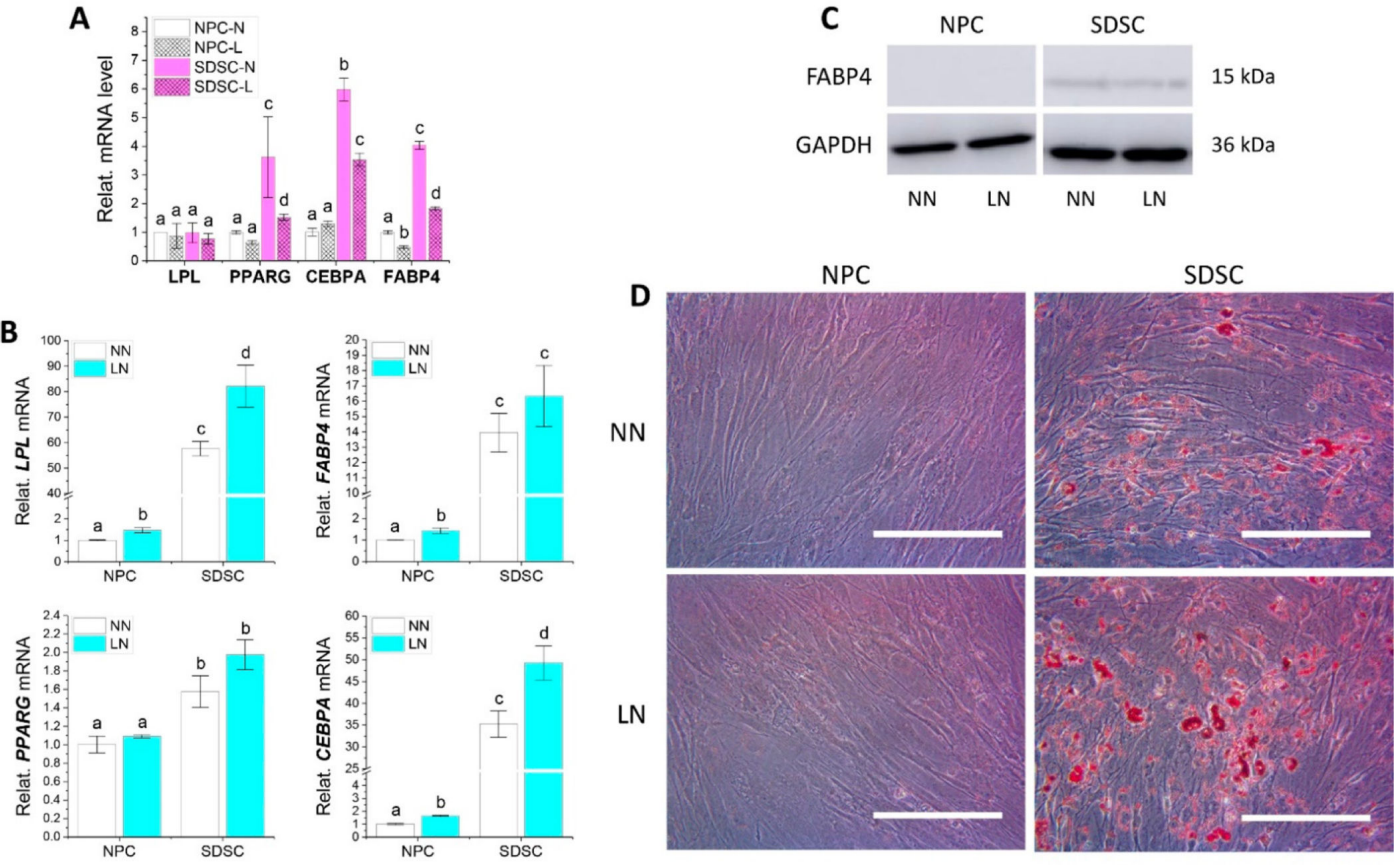
Evaluation of the effect of hypoxia pretreatment on adipogenic potential of fetal MSCs. Fetal NPCs and SDSCs after normoxia (N) or hypoxia treatment (L) were incubated for 21 days in adipogenic induction medium. Adipogenic marker genes *LPL*, *FABP4*, *PPARG*, and *CEBPA* were assessed using RT-qPCR before (**A**: cell sample) and after induction (**B**: day 21 samples), normalized against *GAPDH* levels as an internal control. Data (*n* = 4) are represented in bar charts. Different letters indicate a statistically significant difference compared to the groups within the same gene type (*p* < 0.05). Western blot was used to detect FABP4 expression (**C**) in fetal MSCs after adipogenic induction. GAPDH was used as an internal control. Oil Red O (ORO) staining was used to detect lipid droplets (**D**).

To determine whether hypoxia pretreatment had any influence on adipogenic differentiation of both fetal cell types, RT-qPCR, western blot, and ORO staining were used for evaluation after 21-day adipogenic induction. We found that hypoxia decreased adipogenic marker gene expression including *FABP4* and *PPARG* in both fetal cells as well as *CEBPA* of SDSCs (Figure 5A). However, hypoxia pretreated fetal cells significantly increased *LPL* and *CEBPA* expression in both fetal cells as well as *FABP4* expression of NPCs after adipogenic induction (Figure 5B). RT-qPCR also showed that SDSCs exhibited significantly higher adipogenic marker gene expression compared to NPCs (Figure 5B), which was confirmed by FABP4 expression via western blot (Figure 5C) and lipid droplets via ORO staining (Figure 5D) in adipogenically induced SDSCs.

**Figure 6.**
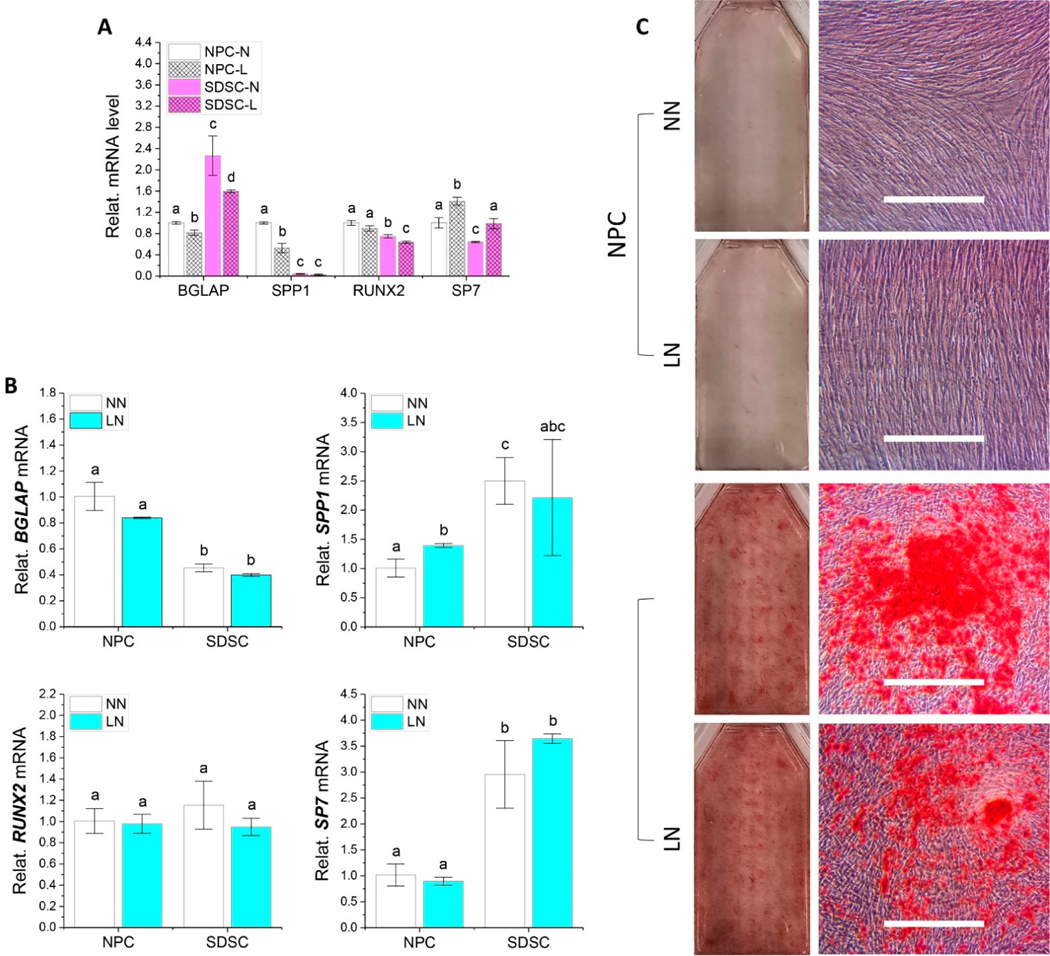
Evaluation of the effect of hypoxia pretreatment on osteogenic potential of fetal MSCs. Fetal NPCs and SDSCs after normoxia (N) or hypoxia treatment (L) were incubated for 21 days in osteogenic induction medium. Osteogenic marker genes *BGLAP*, *SPP1*, *RUNX2*, and *SP7* were assessed using RT-qPCR before (**A**: cell sample) and after induction (**B**: day 21 samples), normalized against *GAPDH* levels as an internal control. Data (*n* = 4) are represented in bar charts. Different letters indicate a statistically significant difference compared to the groups within the same gene type (*p* < 0.05). Alizarin Red S (ARS) staining was used to detect bone nodules (**C**).

To determine whether hypoxia pretreatment had any influence on osteogenic differentiation of both types of fetal cells, RT-qPCR and ARS staining were used for evaluation after 21-day osteogenic induction. We found that hypoxia decreased gene expression of the osteogenic marker *BGLAP* but increased *SP7* in both fetal cell types as well as decreased *SPP1* of NPCs and *RUNX2* in SDSCs (Figure 6A). After osteogenic induction, hypoxia pretreatment did not make a significant difference in osteogenic marker gene expression of both fetal cells; however, different from the higher expression of *BGLAP* in NPCs, SDSCs exhibited higher levels of *SPP1* and *SP7* (Figure 6B), which was confirmed by ARS staining for calcium deposits (Figure 6C).

### 3.4. Effects of Hypoxia on Chondrogenic Differentiation of Fetal Cells

**Figure 7.**
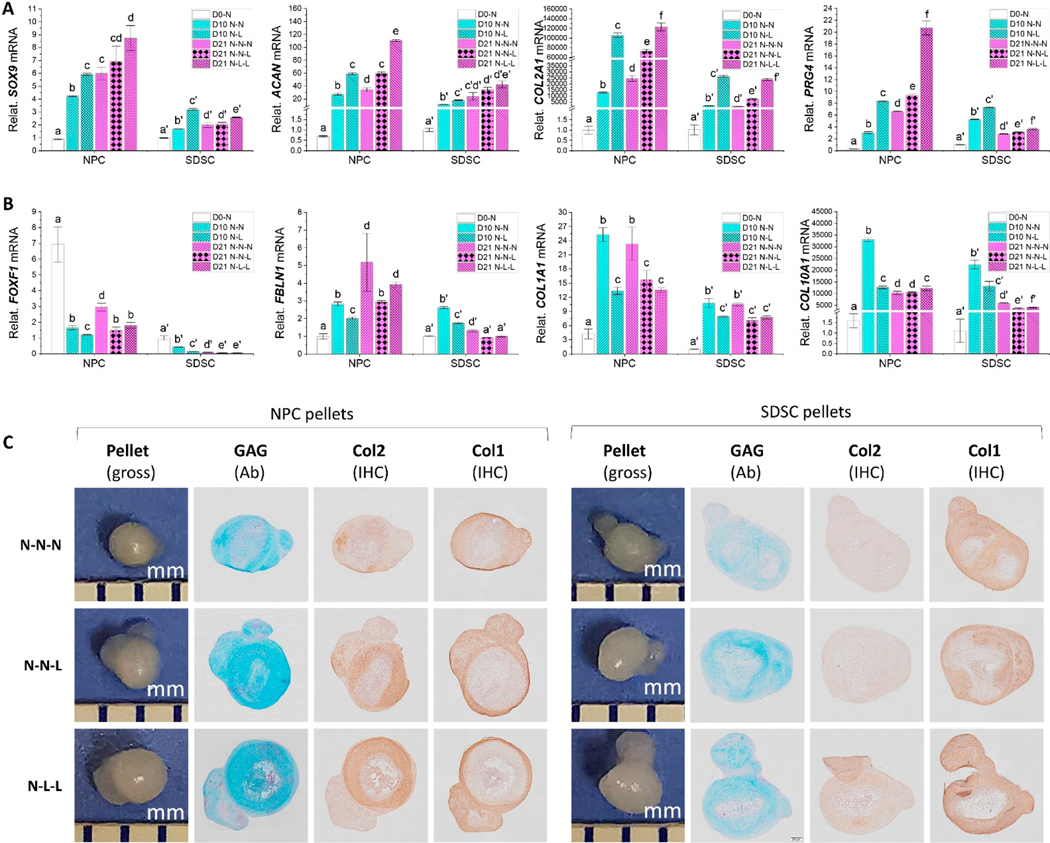
Evaluation of the effect of hypoxia on chondrogenic differentiation of fetal MSCs. RT-qPCR was used to assess the expression of chondrogenic marker genes (*SOX9*, *ACAN*, *COL2A1*, and *PRG4*) (**A**) and chondrogenic related genes (*FOXF1*, *FBLN1*, *COL1A1*, and *COL10A1*) (**B**) in days 0, 10, and 21 pellets, normalized against *GAPDH* levels as an internal control. Data (*n* = 4) are represented in bar charts. Different letters indicate a statistically significant difference compared to the group within the same cell type (*p* < 0.05). Histology (**C**) using Alcian blue staining (Ab) for sulfated GAG and immunohistochemical staining (IHC) for type II collagen (Col2) and type I collagen (Col1) along with a gross photo to indicate the size of pellet (mm) in day 21 chondrogenically induced pellets.

To determine the direct effect of hypoxia on chondrogenic differentiation, after ex vivo expansion in normoxia, fetal cells were cultured in a pellet system in either normoxia (N-N-N), hypoxia (N-L-L), or partial hypoxia (N-N-L) for 21 days. We found that the N-L-L pellets exhibited the highest expression levels of chondrogenic marker genes including *SOX9*, *ACAN*, *COL2A1*, and *PRG4* (Figure 7A). Despite comparable expression of *SOX9*, *ACAN*, and *COL2A1* in day 0 NPC and SDSC pellets, NPC pellets exhibited a greater level of expression at both day 10 and day 21 than the SDSC counterparts, particularly for hypoxia treated pellets (Figure 7A). Intriguingly, despite a lower level of *PRG4* expression in day 0 NPC pellets, 21-day chondrogenic induction dramatically increased its expression in NPC pellets compared to the SDSC counterpart, particularly for hypoxia-treated pellets (Figure 7A).

Interestingly, we found another two related markers *FOXF1* and *FBLN1*, previously demonstrated to be primarily in nucleus pulposus and articular cartilage, respectively [22], responded to hypoxia differently from the above chondrogenic gene expression. Despite higher expression of *FOXF1* in day 0 NPC pellets compared to SDSC pellets, chondrogenic induction dramatically decreased its expression at day 10 followed by an increase at day 21 in NPC pellets while SDSC pellets exhibited a continued decrease for up to 21 days (Figure 7B). We also found that day 0 NPC pellets exhibited comparable expression of *FBLN1* to SDSC pellets (Figure 7B) despite higher levels in SDSC versus NPC cell samples (Figure 3C). Despite the fact that chondrogenic induction favored *FBLN1* expression in day 21 NPC pellets, hypoxia decreased expression of *FOXF1* and *FBLN1* as well as *COL1A1* and *COL10A1* in both NPC and SDSC pellets under chondrogenic induction (Figure 7B).

After 21-day chondrogenic induction, the L-L-L pellets showed the largest size and the most intensity of Alcian blue staining for sulfated GAGs and immunohistochemical staining for type II collagen whereas the N-N-N pellets displayed the most intensity of immunohistochemical staining for type I collagen (Figure 7C), which is in line with the RT-qPCR results (Figure 7A,B). We also found that SDSC pellets showed less intensity of both sulfated GAGs and type II collagen staining (Figure 7C).

### 3.5. Effects of Hypoxia on Adipogenic and Osteogenic Differentiation of Fetal Cells

**Figure 8.**
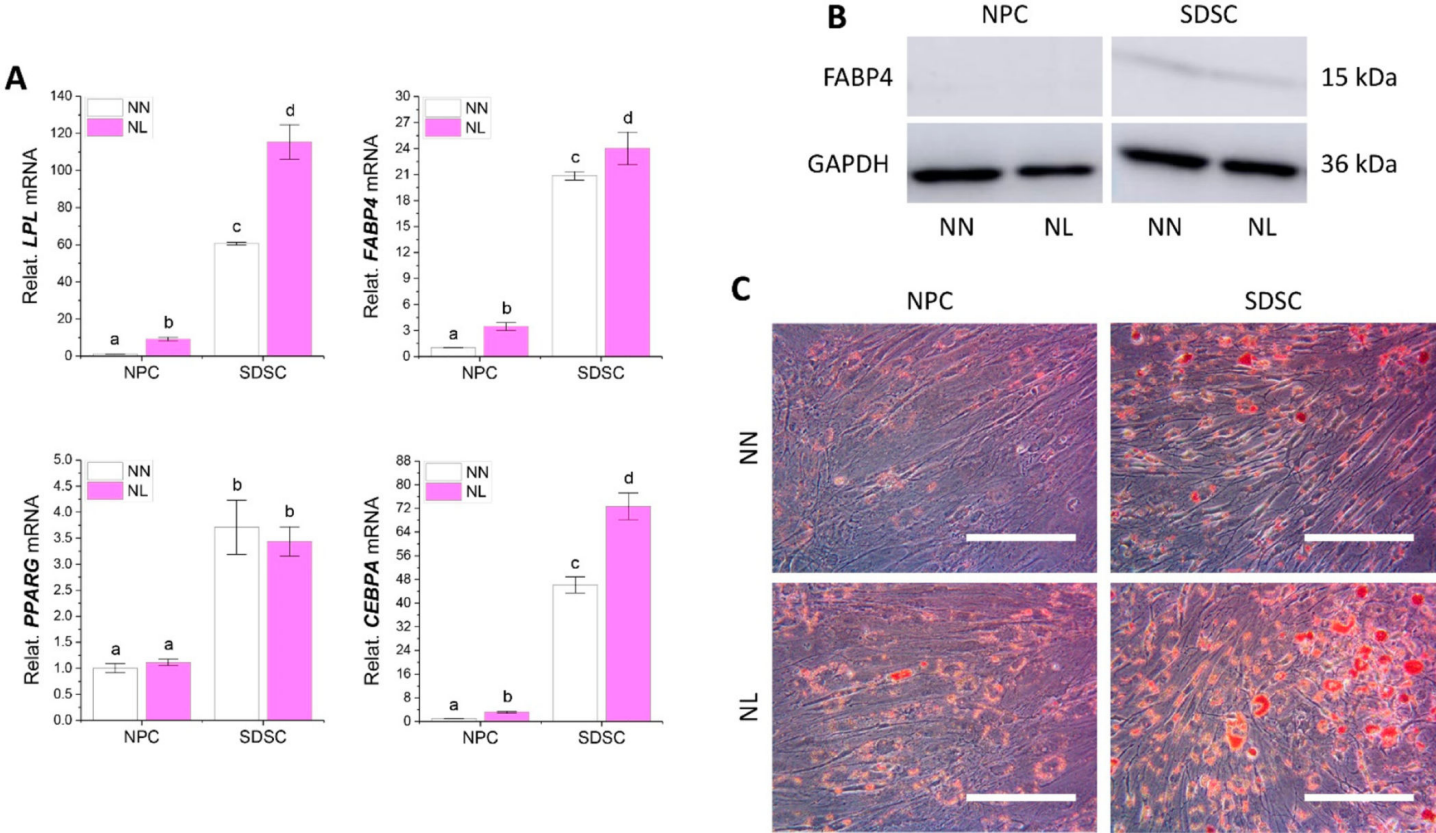
Evaluation of the effect of hypoxia on adipogenic differentiation of fetal MSCs. Normoxia expanded NPCs and SDSCs were incubated in adipogenic medium for 21 days in either normoxia (NN) or hypoxia (NL). RT-qPCR was used to evaluate the expression of adipogenic marker genes (*LPL*, *CEBPA*, *FABP4*, and *PPARG*) (**A**), normalized against *GAPDH* levels as an internal control. Data (*n* = 4) are represented in bar charts. Different letters indicate a statistically significant difference compared to the group within the same cell type (*p* < 0.05). Western blot was used to assess the expression of the FABP4 protein (**B**). GAPDH served as an internal control. Oil Red O (ORO) staining was used to stain lipid droplets (**C**).

To determine the direct effect of hypoxia on adipogenic differentiation, after ex vivo expansion in normoxia, fetal cells were cultured in T25 flasks in either normoxia (NN) or hypoxia (NL) for 21-day adipogenic induction. We found that hypoxia dramatically increased expression levels of adipogenic marker genes including *LPL*, *FABP4*, and *CEBPA* in both NPCs and SDSCs (Figure 8A). NPCs were less responsive to hypoxia compared to SDSCs (Figure 8A), which was supported by western blot results, in which FABP4 was highly expressed in adipogenically induced SDSCs (Figure 8B). ORO staining data indicated that more lipid droplets were found in adipogenically induced SDSCs compared to NPCs and fetal cells treated with hypoxia compared to normoxia (Figure 8C), which further confirmed the mRNA data (Figure 8A).

**Figure 9.**
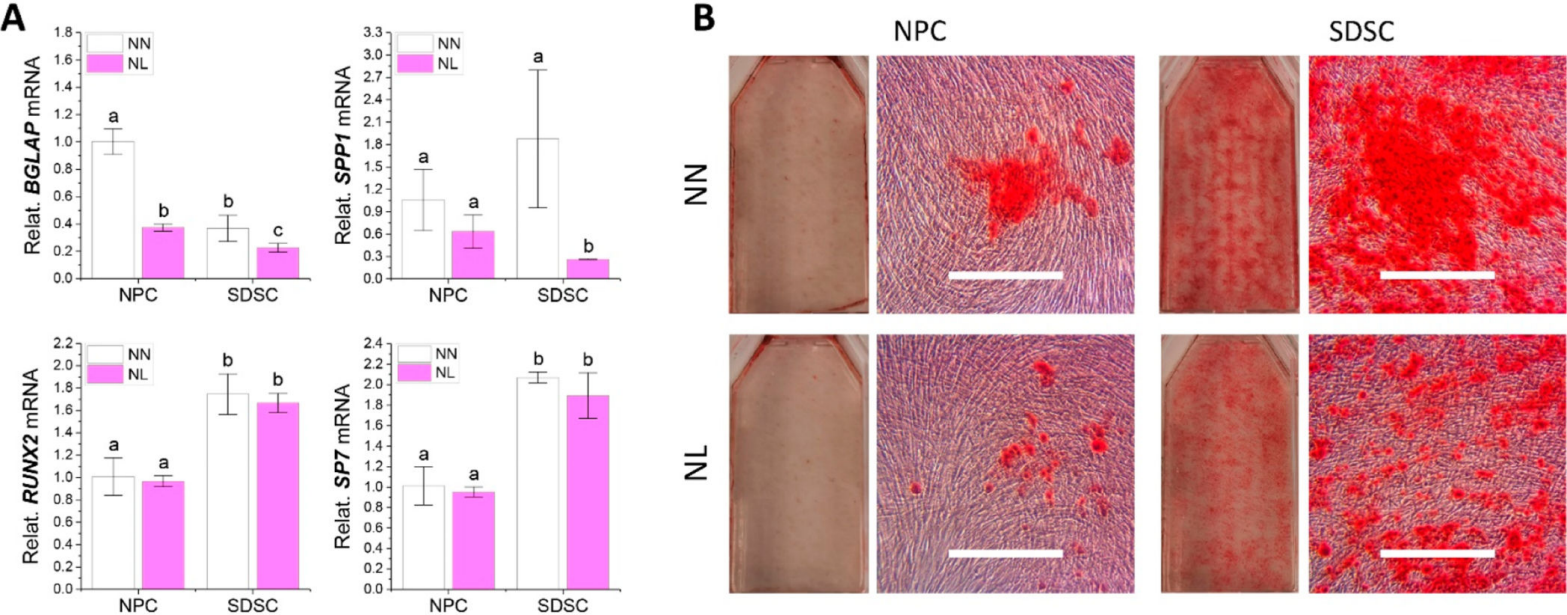
Evaluation of the effect of hypoxia on osteogenic differentiation of fetal MSCs. Normoxia expanded NPCs and SDSCs were incubated in osteogenic medium for 21 days in either normoxia (NN) or hypoxia (NL). RT-qPCR was used to assess expression of osteogenic marker genes (*BGLAP*, *RUNX2*, *SPP1*, and *SP7*) (**A**), normalized against *GAPDH* levels as an internal control. Data (*n* = 4) are represented in bar charts. Different letters indicate a statistically significant difference compared to the group within the same cell type (*p* < 0.05). Matrix mineralization was evaluated using Alizarin Red S (ARS) staining for calcium deposition (**B**).

To determine the direct effect of hypoxia on osteogenic differentiation, after ex vivo expansion in normoxia, fetal cells were cultured in T25 flasks in either normoxia (NN) or hypoxia (NL) for 21-day osteogenic induction. We found that hypoxia decreased expression levels of osteogenic marker genes including *BGLAP* and *SPP1* in both NPCs and SDSCs; intriguingly, no significant difference was found in *RUNX2* and *SP7* expression in both cell types (Figure 9A). ARS staining also confirmed that hypoxia decreased bone nodules in both cell types (Figure 9B).

## 4. Discussion

Compared to adult stem cells, which have been well-studied in regard to the hypoxic influence on both proliferation and differentiation, few reports have focused on fetal stem cells. The objective of this study was to characterize the regenerative potential of fetal cells under hypoxia. Due to the importance of hypoxia in cartilage development and regeneration, two fetal cell sources were chosen for chondrogenic evaluation based on their associations with cartilage. Given their tremendous regenerative potential, both fetal cell types were also evaluated for their adipogenic and osteogenic potential/differentiation under hypoxia. We found that hypoxia during both cell expansion and differentiation phases increased chondrogenic potential and differentiation, respectively. Interestingly, while adipogenic capacity was increased upon both hypoxic expansion and differentiation, osteogenic capacity was basically unchanged following hypoxic expansion and decreased upon hypoxic differentiation.

We found hypoxia increased cell proliferation and expression of MSC surface markers, suggesting that hypoxia helps to maintain fetal cell plasticity and suppress random differentiation, similar to previous studies in adult MSCs in which hypoxia preconditioning prevented cell senescence while enhancing proliferation, differentiation, and migration [9,25,26].

Our data suggested that hypoxic pretreatment not only significantly increased chondrogenic marker gene expression but also had the lowest levels of types I and X collagen expression in both fetal MSCs, which indicates that hypoxia preconditioning facilitates cartilage phenotypic stability and maturation. This finding aligns with a previous report that hypoxia has an anabolic effect on adult MSC chondrogenesis that upregulates chondrogenic genes and downregulates hypertrophic genes [27]. Potential mechanisms associated with hypoxia signals were demonstrated through hypoxia transcription factors (HIFs), particularly HIF1α and HIF2α, and the PI3K/Akt/FoxO pathways [16]. These pathways help to mediate cellular responses to oxygen levels and regulate inflammatory cytokines. For instance, when evaluating hypoxic prechondrogenic cells in mice, HIF1α activity has been linked to a two-fold increase of *SOX9* expression in hypoxic conditions when compared to normoxic conditions [28]. In human articular chondrocytes, *HIF2α* knockout has been shown to decrease chondrogenic gene expression [29].

We found that although both cell types demonstrated increased chondrogenic potential in response to hypoxic pretreatment, the two fetal cell types displayed different sensitivities to chondrogenic induction, with NPCs displaying the more intense response. When comparing fetal NPC and SDSC responses to low oxygen, NPCs appear to be more sensitive to low oxygen, as they exhibited increasing gene expression going from day 10 to day 21 pellets. In contrast, SDSC day 10 pellets showed high chondrogenic differentiation marker expression, but then demonstrated a slight downward trend in going from day 10 to day 21 pellets. Our finding is consistent with previous reports comparing human adult NPCs and other sources of MSCs; for instance, adult NPCs have previously been compared to adult bone marrow-derived MSCs (BMSCs) in regard to their chondrogenic, adipogenic, and osteogenic differentiation potentials, where they demonstrated similarly higher chondrogenic differentiation capacity [30]. Interestingly, we found that chondrogenic induction with hypoxia treatment, either in the early stage (N-L-N) or in the whole differentiation stage (N-L-L), yielded 21-day pellets with the highest expression level of *PRG4* in both fetal cell groups. Given that lubricin is a secreted protein that helps in joint lubrication and movement, 21-day NPC pellets with a higher expression level of *PRG4* compared to SDSC counterparts suggest that fetal NPCs might be a better cell source for articular cartilage regeneration.

Furthermore, we found that both NPC normoxia and hypoxia expanded cell samples showed significantly higher expression levels of condensation marker genes *CDH2* and *FN1* than SDSC normoxia and hypoxia expanded cell samples. Given that cell condensation is an integral component of chondrogenesis [23,24], as indicated by the higher *CDH2* and chondrogenic marker gene expression relative to corresponding SDSC pellets despite a smaller pellet size, NPCs likely form denser chondrogenic pellets than SDSCs. This characteristic is expected as NPCs are more closely related to chondrocytes in the undifferentiated state.

However, even though NPCs share a common phenotype with articular cartilage cells along with similar expression of COL2A1, ACAN, and SOX9, NPCs display morphologic and physiologic differences and can be distinguished using gene expression. FOXF1 has previously been identified as a potential human NPC marker as it displays the highest expression levels in NPCs in comparison to other cell sources [22]. In contrast, FBLN1 is an articular cartilage marker and a negative NP marker [31]. Accordingly, our data indicated that D0 SDSC pellets had higher *FBLN1* expression than their NPC counterparts, which is reasonable since SDSCs are more closely related to articular cartilage. Later on, during chondrogenic induction, NPC pellets displayed a large increase in *FBLN1* levels, suggesting characteristic changes toward an articular cartilage-like phenotype. This finding was also confirmed by *FOXF1* levels, which were initially more highly expressed in NPCs but lowered in expression during differentiation.

Interestingly, while our data show that NPCs demonstrate greater chondrogenic potential than SDSCs, SDSCs showed greater adipogenic and osteogenic potentials. These results are mirrored by a previous study comparing fetal spine cells and adult BMSCs which found fetal spine cells displayed lower adipogenic and osteogenic potential [32]. In BMSCs, hypoxia has been shown to enhance MSC proliferation and osteogenesis, likely through growth factor production [33]. However, as an intervertebral disc cell, NPCs have been clinically observed to hinder disc calcification, likely due to NPC secretion of bone morphogenetic protein antagonists, such as chordin, gremlin 1, and noggin, that inhibit osteogenic differentiation [34].

In our study, we found that hypoxic expansion and differentiation increased adipogenic capacity. The finding aligns with reports that hypoxic exposure during expansion enhances adipose-derived MSC adipogenic potentials [35]. Certain studies have found that hypoxia (1–2%) can trigger reactive oxygen species generation, thus inducing adipogenesis [36,37]. This increase in adipogenesis is further observed in BMSCs under extreme hypoxia (0.2%) [38].

It should be noted that in this study, we utilized human fetal stem cells. Unlike human adult stem cells, human fetal stem cells exist in an inherently different environment in which, while the overall fetal physiological environment is slightly more hypoxic than the adult physiological environment, fetal immature cartilage typically has blood vessels. Compared to adult MSCs, fetal MSCs have shown greater self-renewal capacity and multilineage potential, which make them good prospective candidates for cell-based therapies. Furthermore, fetal cells have low immunogenicity as they express lower levels of human leukocyte antigen (HLA) class I and do not express HLA class II [39,40], thus making them more suitable candidates for therapeutic transplantation. Compared to embryonic stem cells, they are also less ethically controversial.

A limitation of this study is that the culture system is not perfect. We used a standard incubator of 20% oxygen and 5% carbon dioxide to represent “normoxia”; however, we should note that 20% oxygen is higher than most cell physiological environments [41]. It should also be specified that the “hypoxia” condition was achieved when the cells were incubated in a low oxygen incubator; during medium changes, the cells would have been temporarily exposed to normoxic conditions.

In conclusion, although hypoxic preconditioning led to superior chondrogenic potential, it is worth noting that hypoxic differentiation resulted in stronger chondrogenic gene expression. Thus, the combination of hypoxic conditioning pretreatment and differentiation could hold great potential for epigenetically programming chondrocytes prior to clinical implantation. NPCs’ predisposition toward chondrogenesis and inhibition of osteogenesis makes them the ideal candidate for cartilage tissue engineering. As we saw, NPCs are capable of closely mimicking chondrocyte condensation, phenotypic composition, and gene expression. These factors make it a more phenotypically stable cell source than SDSCs.

## Figures and Tables

**Table 1.** Target genes’ Assay ID information for RT-qPCR.

Gene Name	Full Name	Assay ID
	Stemness related genes	
BMI1	B lymphoma Mo-MLV insertion region 1 homolog	Hs00180411_m1
KLF4	Kruppel-like factor 4	Hs00358836_m1
MYC	MYC proto-oncogene	Hs00153408_m1
NANOG	Nanog Homeobox	Hs02387400_g1
NES	Nestin	Hs04187831_g1
NOV	Nephroblastoma overexpressed	Hs00159631_m1
POU5F1	POU class 5 homeobox 1	Hs04260367_gH
SOX2	SRY-box 2	Hs01053049_s1
	Chondrogenesis related genes	
*ACAN*	Aggrecan	Hs00153936_m1
*CDH2*	Cadherin 2	Hs00983056_m1
*COL2A1*	Type II collagen	Hs00156568_m1
*COL1A1*	Type I collagen	Hs00164004_m1
*COL10A1*	Type X collagen	Hs00166657_m1
*PRG4*	Proteoglycan 4	Hs00981633_m1
*SOX9*	SRY-Box 9	Hs00165814_m1
	Adipogenesis related genes	
*CEBPA*	CCAAT/enhancer-binding protein alpha	Hs00269972_s1
*FABP4*	Fatty acid-binding protein 4	Hs01086177_m1
*LPL*	Lipoprotein lipase	Hs00173425_m1
*PPARG*	Peroxisome Proliferator Activated Receptor Gamma	Hs01115513_m1
	Osteogenesis related genes	
*BGLAP*	Osteocalcin	Hs01587814_g1
*SPP1*	Osteopontin	Hs00959010_m1
*SP7*	Osterix	Hs01866874_s1
*RUNX2*	Runt-related transcription factor 2	Hs00231692_m1
	Other related genes	
*FBLN1*	Fibulin 1	Hs00972609_m1
*FN1*	Fibronectin 1	Hs01549976_m1
*FOXF1*	Forkhead Box F1	Hs00230962_m1
*GAPDH*	Glyceraldehyde-3-phosphate dehydrogenase	Hs02758991_g1
*HSPG2*	Heparan Sulfate Proteoglycan 2	Hs01078536_m1

## Data Availability

Not applicable.
